# Noninvasive Mechanical Joint Loading as an Alternative Model for Osteoarthritic Pain

**DOI:** 10.1002/art.40835

**Published:** 2019-05-17

**Authors:** Freija ter Heegde, Ana P. Luiz, Sonia Santana‐Varela, Iain P. Chessell, Fraser Welsh, John N. Wood, Chantal Chenu

**Affiliations:** ^1^ Royal Veterinary College and University College London London UK; ^2^ University College London London UK; ^3^ AstraZeneca Cambridge UK; ^4^ Royal Veterinary College London UK

## Abstract

**Objective:**

Mechanisms responsible for osteoarthritic (OA) pain remain poorly understood, and current analgesic therapies are often insufficient. This study was undertaken to characterize and pharmacologically test the pain phenotype of a noninvasive mechanical joint loading model of OA, thus providing an alternative murine model for OA pain.

**Methods:**

The right knees of 12‐week‐old male C57BL/6 mice were loaded at 9N or 11N (40 cycles, 3 times per week for 2 weeks). Behavioral measurements of limb disuse and mechanical and thermal hypersensitivity were acquired before mechanical joint loading and monitored for 6 weeks postloading. The severity of articular cartilage lesions was determined postmortem with the Osteoarthritis Research Society International scoring system. To assess efficacy of various treatments for pain, 9N‐loaded mice were treated for 4 weeks with diclofenac (10 mg/kg), gabapentin (100 mg/kg), or anti–nerve growth factor (anti‐NGF) (3 mg/kg).

**Results:**

Mechanical hypersensitivity and weight bearing worsened significantly in 9N‐loaded mice (n = 8) and 11N‐loaded mice (n = 8) 2 weeks postloading, compared to baseline values and nonloaded controls. Maximum OA scores of ipsilateral knees confirmed increased cartilage lesions in 9N‐loaded mice (mean ± SEM 2.8 ± 0.2; *P* < 0.001) and 11N‐loaded mice (5.3 ± 0.3; *P* < 0.001), compared to nonloaded controls (1.0 ± 0.0). Gabapentin and diclofenac restored pain behaviors to baseline values after 2 weeks of daily treatment, and gabapentin was more effective than diclofenac. A single injection of anti‐NGF alleviated nociception 2 days after treatment and remained effective for 2 weeks, with a second dose inducing stronger and more prolonged analgesia.

**Conclusion:**

Our findings show that mechanical joint loading induces OA lesions in mice and a robust pain phenotype that can be reversed using analgesics known to alleviate OA pain in patients. This establishes the use of mechanical joint loading as an alternative model for the study of OA pain.

## Introduction

Osteoarthritis (OA) is a common degenerative joint disease associated with chronic, debilitating pain in the affected joints which significantly reduces mobility and quality of life in patients 1. Current therapies used to treat OA pain are often insufficient, including nonsteroidal antiinflammatory drugs (NSAIDs), which can produce unwanted side effects that limit long‐term use 2. OA pathology and progression have been examined in detail, but mechanisms contributing to OA pain and the relationship between pain and OA pathology remain poorly understood. Therefore, there is a need for a well‐characterized, noninvasive murine model of OA pain that exhibits both a robust, reproducible pain phenotype and histologic evidence of OA pathology.

The 2 most commonly used OA models in the preclinical field of OA pain are the monosodium iodoacetate (MIA) model used to induce inflammatory OA 3 and surgical destabilization of the joint typically used to model posttraumatic OA 4, 5. In the MIA model, a single intraarticular injection of MIA is administered in the knee joint and inhibits the glycolytic pathway, causing chondrocytic cell death and an acute inflammation leading to cartilage erosion and joint disruption 6, 7. The MIA injection causes immediate onset of mechanical hyperalgesia 8, 9, altered weight bearing 10, and reduction in mobility 11, all of which are associated with the early inflammatory phase (days 0–7) of OA. This is then followed by a more persistent allodynia, typical in late‐phase OA (days 14–28). Pain‐like behaviors increase in a dose‐dependent manner, with late‐phase hypersensitivity typically observed with higher doses of MIA 12. Surgical models, such as destabilization of the medial meniscus (DMM) 4 or partial medial meniscectomy (PMM) 5, 13, are used predominantly in mice and rely on the surgical destabilization of the medial meniscus, which typically leads to cartilage damage 4–8 weeks postsurgery 5, 14, 15. Pain‐like behaviors typically take longer to develop: mechanical hypersensitivity can develop 4 weeks postsurgery, a decrease in spontaneous naturalistic behaviors has been seen 8 weeks postsurgery, and altered weight bearing has been observed as late as 12 weeks postsurgery 13, 16, 17. Mice undergoing sham surgery also show significant amounts of postsurgical pain 16, 17, with pain thresholds not returning to baseline levels for as long as 8 weeks 18. A major drawback of both models is the invasiveness of the procedures, which adds a layer of joint disruption that influences both joint damage and the resulting pain behaviors in affected and sham‐operated animals.

The noninvasive mechanical joint loading model was initially used to investigate the osteogenic effect of mechanical loading on bone 19 and has recently been adapted to investigate the pathogenesis of OA 20. In this model, OA is induced through intermittent, repetitive loading of the tibia through the knee and ankle joints. Histologic cartilage changes have been characterized in mice and show that single loading episodes induce lesions in the articular cartilage 20. When loading episodes are repeated 3 times weekly for 2 weeks, these lesions spontaneously progress and worsen over a period of 3 weeks 20. This model also shows changes in the subchondral bone 21 that are consistent with pathology seen in humans. Because of the relatively recent introduction of mechanical joint loading as a model for OA, the pain phenotype in this model has not yet been fully characterized.

The aim of this study was to characterize the pain phenotype in the murine mechanical joint loading model of OA to determine if it can be used as a model for OA pain. To this end, we induced OA of different severities using 2 load magnitudes and monitored hypersensitivity thresholds over time using an array of established behavioral assays developed in mice 22. The presence of OA knee pathology was confirmed at the end of the study by quantifying cartilage damage. Furthermore, we investigated whether diclofenac, gabapentin, or anti–nerve growth factor (anti‐NGF) monoclonal antibody (mAb) could alleviate the OA pain seen in this model. Diclofenac is an NSAID that is effective against inflammatory pain and the first‐line treatment for patients with OA pain 23, while gabapentin is an antiepileptic drug that is effective in treating complex neuropathic pain syndromes 24, 25. Anti‐NGF antibodies represent novel analgesics currently in clinical trials for OA pain 26, 27, 28. In vivo studies show that anti‐NGF treatment restores spontaneous day/night activity in mice with orthopedic surgery–induced pain 29 and improves gait imbalance in both the MIA model 30 and a surgical model 31 of OA. Additionally, treatment with the soluble NGF receptor TrkAd5 effectively restored the altered weight bearing seen immediately after DMM surgery (postoperative pain) and 16 days postsurgery (OA pain) 32. Testing the efficacy of these drugs in alleviating pain is the first step in validating mechanical joint loading as an appropriate model for OA pain.

## Materials and Methods

#### Animals

Ten‐week‐old treatment‐naive C57BL/6 mice (Charles River) were housed in groups of 4 in individually ventilated cages and fed a standard RM1 maintenance diet ad libitum. The environment was climate‐ and light‐controlled: temperature 22°C, humidity 50%, lights on 7:00 am–7:00 pm. Animals were acclimatized for 1 week before the start of procedures, which were conducted during the light phase (8:00 am–6:00 pm). All experiments were carried out in compliance with the Animals (Scientific Procedures) Act 1986 and approved by the UK Home Office license.

#### In vivo mechanical joint loading

OA was induced in mice by a 2‐week loading regimen 20 using an electronic materials testing machine (Bose 3100). Mice were 12 weeks old at the start of loading, which was performed under general anesthesia (3.5% isoflurane). The right tibia was positioned vertically between 2 custom‐made loading cups that restricted the knee and ankle joints in deep flexion. Axial compressive loads were applied through the knee joint via the upper loading cup, while a loading cell, attached to the lower cup, registered and monitored the applied loads. One loading cycle consisted of 9.9 seconds holding time with a load magnitude of 2N (load needed to maintain knee position), after which a maximum load of 9N or 11N was applied for 0.05 seconds, with a rise and fall time of 0.025 seconds each. This 10‐second trapezoidal wave loading cycle was repeated 40 times within 1 loading episode. During the loading regimen, this loading episode was repeated 3 times per week for 2 consecutive weeks. The load magnitudes of 9N or 11N were chosen to enable comparisons with previously published work on the loading model 19, 20, 21.

#### Experimental design

##### Pain phenotype after mechanical joint loading

Mice loaded at 9N or 11N (n = 8 per group) underwent behavioral measurements at baseline in the week before loading and were monitored weekly for 6 weeks postloading (see Supplementary Methods, on the *Arthritis & Rheumatology* web site at http://onlinelibrary.wiley.com/doi/10.1002/art.40835/abstract). Changes in behavior were compared to changes in age‐ and cage‐matched, nonloaded controls, which were not subjected to any loading regimen but instead underwent isoflurane anesthesia for the same duration as loaded mice. No behavioral testing was performed during the 2 weeks of loading.

##### Pharmacologic validation of mechanical joint loading

The mechanical joint loading model was validated by testing the antinociceptive effect of diclofenac, gabapentin, and an anti‐NGF mAb on 9N‐loaded mice. In total, 6 groups (n = 8 mice per group) were tested: 3 experimental groups in which 1 dose of each drug was tested and 3 control groups (nonloaded saline‐treated, loaded saline‐treated, and loaded inactive control antibody*–*treated mice). Analgesic treatment was administered from week 2 to week 6 weeks postloading. Animals receiving diclofenac (10 mg/kg 33; Sigma‐Aldrich), gabapentin (100 mg/kg 33; Sigma‐Aldrich), or saline (0.9% NaCl; Sigma‐Aldrich) were treated daily via gavage without anesthesia. The volume administered (<500 μl) was calculated according to the weight of the animal. Anti‐NGF mAb treatment (3 mg/kg, intraperitoneally; a generous gift from MedImmune, AstraZeneca) was administered at weeks 2 and 4 postloading. Loaded controls received inactive antibody (3 mg/kg; a generous gift from MedImmune, AstraZeneca) at the same time points.

Pain thresholds were measured at baseline and on a weekly basis after loading in mice receiving saline, diclofenac, or gabapentin. Behavioral testing started 1 hour after treatment. Animals receiving anti‐NGF or inactive antibody were tested 4, 24, and 48 hours and then every 2 days following treatment.

#### Histologic analysis of joints and OA grading

Six weeks postloading, mice were killed by CO_2_ overdose followed by cervical dislocation. Hind limbs were removed and fixed in 4% neutral buffered formalin for 48 hours. Knees were then decalcified (Immunocal; Quartett) for 5 days and processed for paraffin embedding. Once embedded, 6‐μm coronal sequential sections were acquired from the entire joint, of which a quarter was stained with toluidine blue (0.1% in 0.1*M* acetate buffer, pH 5.6). OA severity was scored for each stained section using the Osteoarthritis Research Society International grading system (range 0–6) 34. Briefly, grade 0 = normal surface articular cartilage, grade 0.5 = a loss of toluidine blue staining, grade 1 = lesions in the superficial zone of the articular cartilage, grade 2 = lesions down to the intermediate zone, grade 3 = lesions down to the tidemark with possible loss of articular cartilage from <20% of the surface of the condyle, grade 4 = loss of 20–50% of articular cartilage, grade 5 = loss of 50–80% of articular cartilage, and grade 6 = loss of >80% of articular cartilage and exposure of subchondral bone. For each knee, the maximum OA score, as determined by the lesion with the highest score, and a summed OA score were reported. OA severity was classified as either low (grade 0–2), mild (grade 3–4), or severe (grade 5–6).

#### Statistical analysis

Data were analyzed using GraphPad Prism version 7.04. Results are presented as the mean ± SEM. Mice were assigned conditions in a pseudorandom order, ensuring comparable behavioral baseline values and allocating different conditions within the home cage. Two mice in the diclofenac treatment group were excluded from analysis due to adverse gastrointestinal effects. After confirmation of normal distribution, multiple groups were compared using parametric two‐way analysis of variance, followed by the Bonferroni post hoc test. *P* values less than 0.05 were considered significant.

## Results

#### Induction of chronic mechanical hypersensitivity, altered weight bearing, and reduced mobility by mechanical joint loading

**Figure 1:**
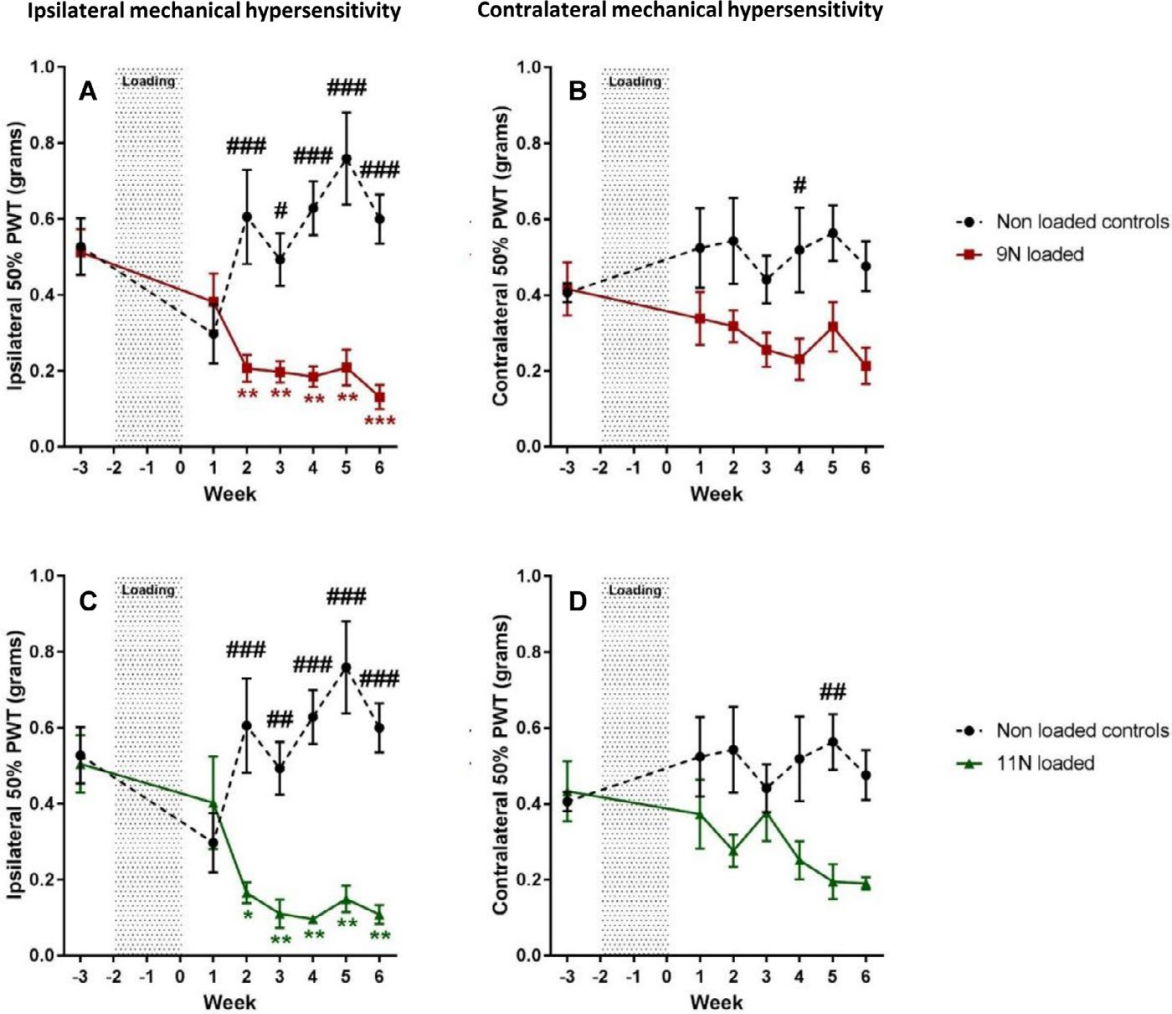
Development of mechanical hypersensitivity after mechanical joint loading. The right knees of mice were loaded 3 times per week for 2 weeks at 9N (n = 8) (**A** and **B**) or 11N (n = 8) (**C** and **D**) to induce osteoarthritis. Development of mechanical hypersensitivity was measured using von Frey filaments (50% paw withdrawal threshold [PWT]) in the ipsilateral paws (**A** and **C**) and contralateral paws (**B** and **D**). Results were compared to those in nonloaded anesthetized controls (n = 8). Values are the mean ± SEM. # = *P* < 0.05; ## = *P* < 0.01; ### = *P* < 0.001, versus mechanical joint loading. * = *P* < 0.05; ** = *P* < 0.01; *** = *P* < 0.001, versus baseline. Color figure can be viewed in the online issue, which is available at http://onlinelibrary.wiley.com/doi/10.1002/art.40835/abstract.

Mechanical joint loading with a load of either 9N or 11N induced a mechanical pain phenotype, which was established 2 weeks postloading and progressively worsened until 6 weeks postloading. From 2 to 6 weeks postloading, both 9N‐loaded mice (Figure 1A) and 11N‐loaded mice (Figure 1C) showed a significant and persistent reduction in ipsilateral mechanical sensitivity, compared to both baseline values and findings in nonloaded controls (*P* < 0.001). Mice loaded with 9N showed a reduction in mechanical sensitivity from baseline (mean ± SEM 0.513 ± 0.06 gm) to 2 weeks postloading (0.207 ± 0.04 gm), with thresholds progressively lowering until 6 weeks postloading (0.131 ± 0.03 gm). Mice loaded with 11N showed a similar trend, with baseline mechanical threshold (0.505 ± 0.08 gm) decreasing 2 weeks postloading (0.165 ± 0.03 gm) and stabilizing up to 6 weeks postloading (0.108 ± 0.03 gm). Notably, there was also a reduction in the mechanical sensitivity thresholds of the contralateral paw, although this developed at a later stage and was not as pronounced as in the ipsilateral paw (Figures 1B and D).

**Figure 2:**
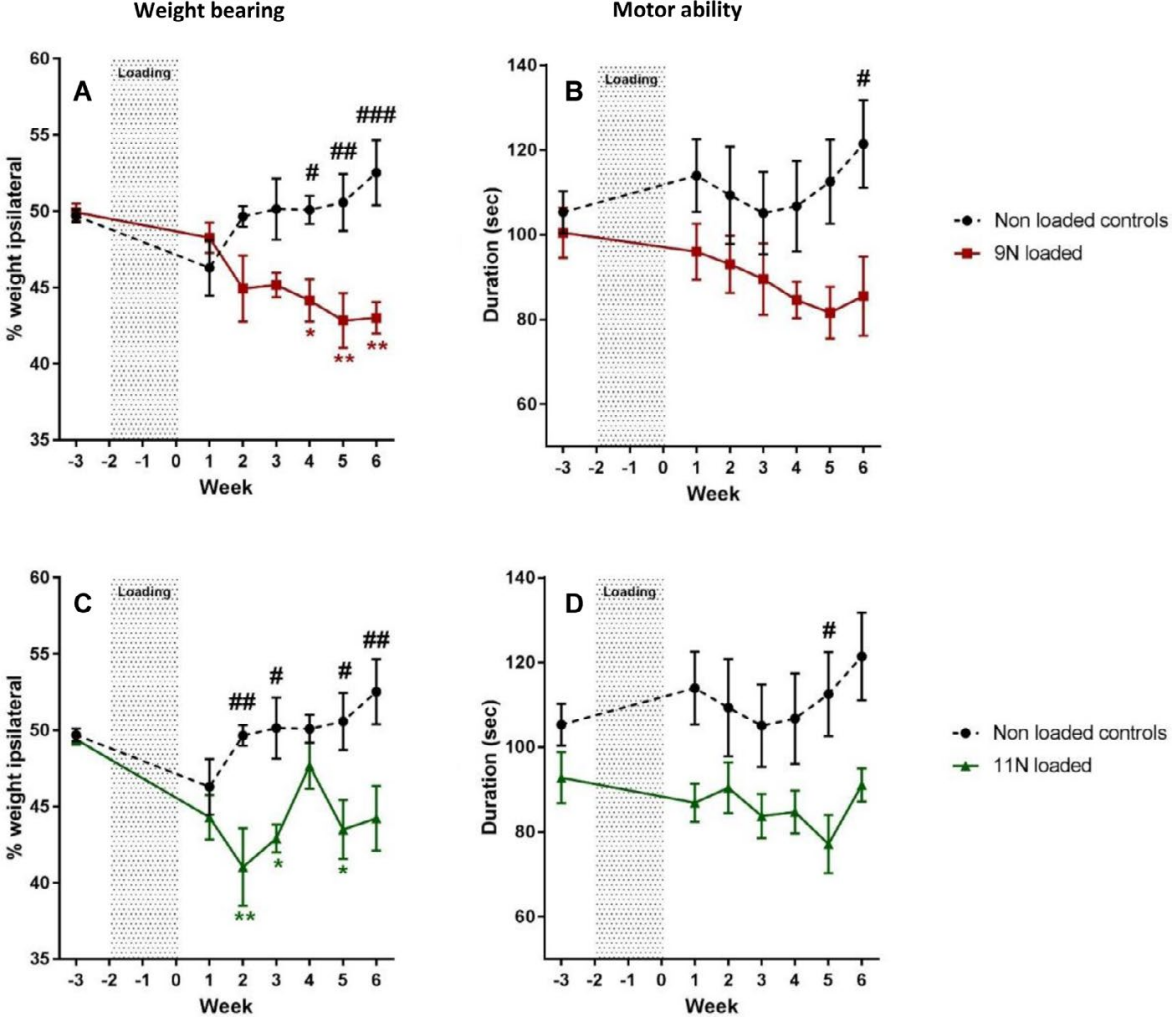
Altered weight bearing and reduced mobility after mechanical joint loading. The right knees of mice were loaded 3 times per week for 2 weeks at 9N (n = 8) (**A** and **B**) or 11N (n = 8) (**C** and **D**) to induce osteoarthritis. Altered weight bearing was assessed using the incapacitance test, which measured the percentage of weight borne on the ipsilateral hind paw (**A** and **C**). Motor ability (**B** and **D**) was measured as the duration mice were able to remain on the rotarod. Results were compared to those in nonloaded anesthetized controls (n = 8). Values are the mean ± SEM. # = *P* < 0.05; ## = *P* < 0.01; ### = *P* < 0.001, versus mechanical joint loading. * = *P* < 0.05; ** = *P* < 0.01, versus baseline. Color figure can be viewed in the online issue, which is available at http://onlinelibrary.wiley.com/doi/10.1002/art.40835/abstract.

The development of mechanical hypersensitivity was accompanied by altered weight bearing and reduction in mobility. Mice loaded with 9N showed a progressive reduction in the percentage of weight borne on the ipsilateral hind limb between baseline (mean ± SEM 49.94 ± 0.6%) and 4 weeks postloading (44.15 ± 1.4%), which was significantly different from weight bearing values in nonloaded controls (*P* = 0.0024) (Figure 2A). Mice loaded with 11N also showed a significant decrease in ipsilateral weight bearing over time compared to nonloaded controls (*P* = 0.0343) (Figure 2C). Percentage values decreased between baseline (49.41 ± 0.3%) and 2 weeks postloading (41.03 ± 2.5%), but had returned at 4 weeks postloading (47.67 ± 1.5%) and decreased again at 6 weeks postloading (44.23 ± 2.1%).

Motor ability was slightly reduced in the 9N‐loaded mice, which showed a decline in time spent on the rotarod compared to nonloaded control mice, reaching significance at 6 weeks postloading (*P* = 0.0238) (Figure 2B). A similar decline was observed in 11N‐loaded mice, which reached significance at 5 weeks postloading (*P* = 0.0071) (Figure 2D).

No differences in thresholds of thermal sensitivity between loaded and nonloaded animals were observed, as assessed by the Hargreaves test and cold plantar assay measurements and using a hot plate (50°C and 55°C) and a cold plate (0°C) (data not shown). The nonloaded control group did not show changes in any of the pain measurements over time.

#### Induction of articular cartilage lesions by mechanical joint loading

**Figure 3:**
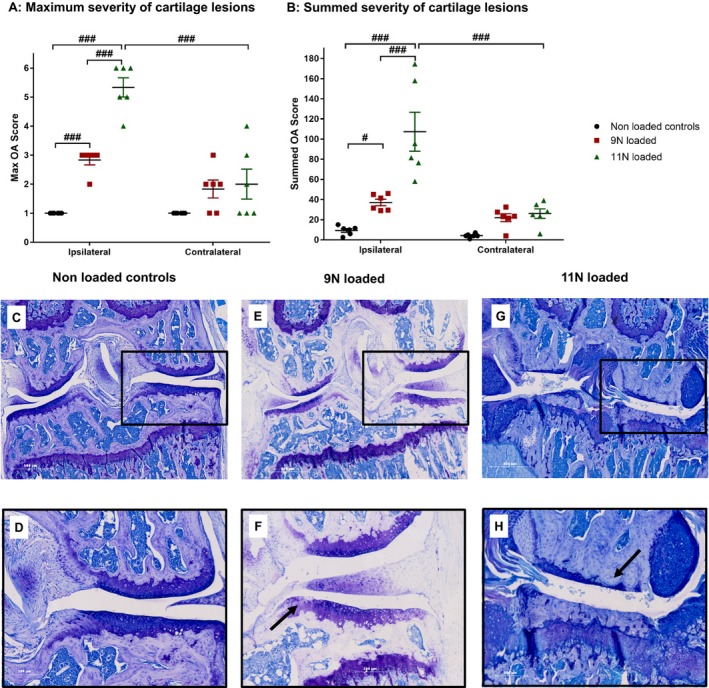
Severity of osteoarthritis (OA) lesions after mechanical joint loading at 9N and 11N. **A** and **B**, Ipsilateral and contralateral knees of nonloaded and loaded mice (9N and 11N) were collected postmortem at 6 weeks postloading. OA severity was scored (range 0–6) as low (0–2), mild (3–4), or severe (5–6). Maximum OA scores (**A**) and summed OA scores (**B**) are shown for nonloaded controls (n = 6), 9N‐loaded mice (n = 6), and 11N‐loaded mice (n = 6). Symbols represent individual mice. Bars show the mean ± SEM. # = *P* < 0.05; ### = *P* < 0.001. **C**–**H**, Examples of typical knee histology of the ipsilateral knee are shown for nonloaded controls (**C** and **D**), 9N‐loaded mice (**E** and **F**), and 11N‐loaded mice (**G** and **H**). **C**,** E**, and **G** show the whole joint. Original magnification × 5. **D**,** F**, and **H** show the medial compartment (boxed areas in **C**,** E**, and **G** at 5 times higher magnification). Arrows show typical cartilage damage seen under each condition.

Histologic analysis of joints revealed that loading at both 9N and 11N induced OA lesions in ipsilateral and contralateral knees, and higher maximum (Figure 3A) and summed (Figure 3B) severity scores compared to the nonloaded controls were observed. Maximum ipsilateral articular cartilage lesion scores were higher in 11N‐loaded mice (mean ± SEM 5.3 ± 0.3) compared to 9N‐loaded mice (2.8 ± 0.2) (*P* < 0.001) (Figure 3A). More information on the development of lesions seen in 9N‐loaded mice at 1, 3, and 6 weeks postloading can be found in Supplementary Data (on the *Arthritis & Rheumatology* web site at http://onlinelibrary.wiley.com/doi/10.1002/art.40835/abstract). Additionally, the contralateral knees showed mild OA lesions in both 9N‐loaded mice (1.8 ± 0.2) and 11N‐loaded mice (2.1 ± 0.5). Due to the extreme OA pathology seen in 11N‐loaded mice compared to that seen in 9N‐loaded mice, a 9N loading regimen was used in the pharmacologic study.

#### Treatment with diclofenac, gabapentin, and anti‐NGF mAb at 2 weeks postloading relieves mechanical hypersensitivity and improves weight distribution without affecting motor ability

**Figure 4:**
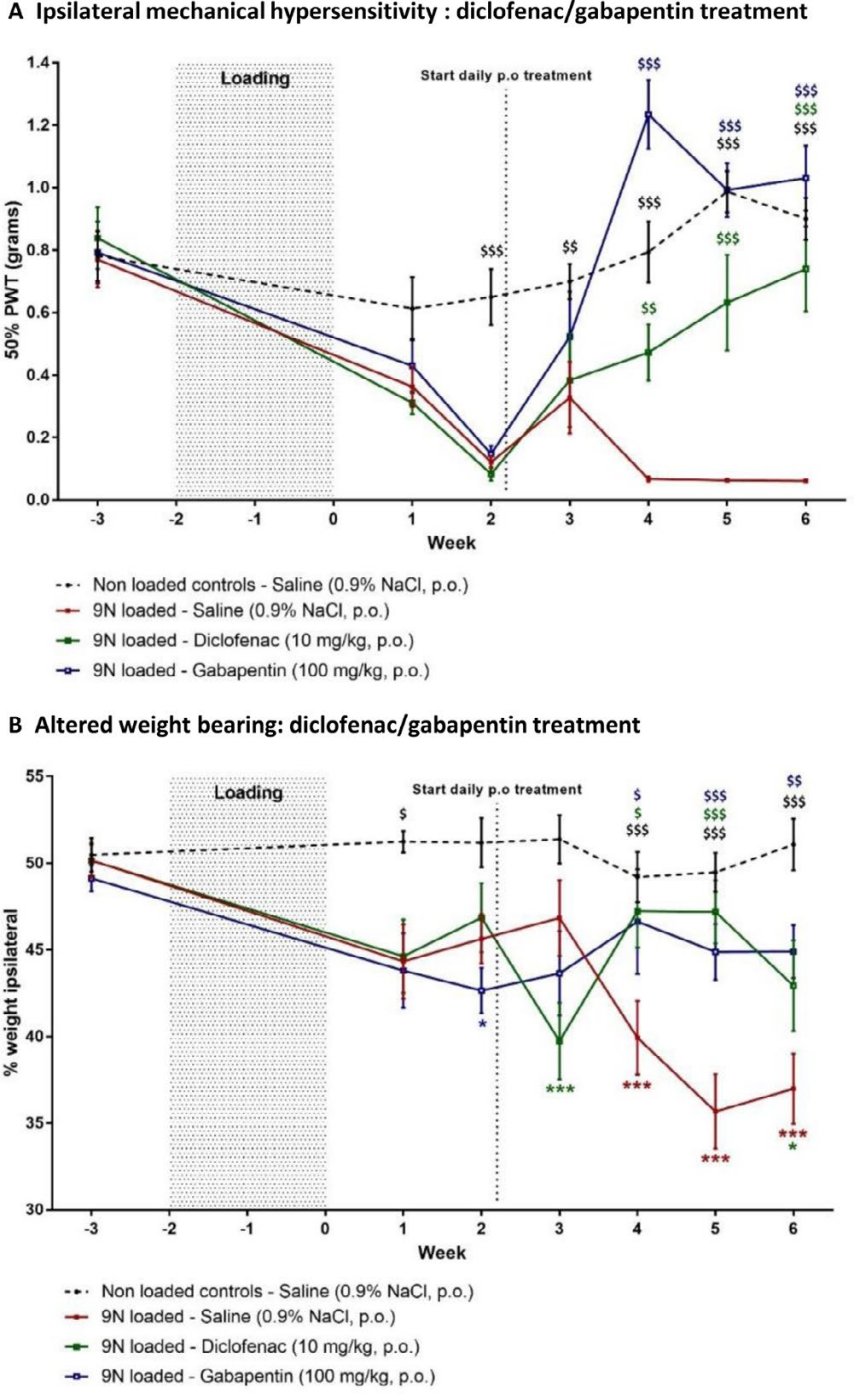
Effect of diclofenac and gabapentin treatment on postloading mechanical hypersensitivity and altered weight bearing. Daily oral (PO) analgesic treatments were started 2 weeks postloading. Mechanical hypersensitivity (**A**), measured as 50% paw withdrawal threshold (PWT), and weight bearing (**B**), measured as the percentage of weight borne on the ipsilateral hind paw, were monitored on a weekly basis in nonloaded and 9N‐loaded animals receiving saline (n = 8 per group), diclofenac (n = 6), or gabapentin (n = 8). Values are the mean ± SEM. $ = *P* < 0.05; $$ = *P* < 0.01; $$$ = *P* < 0.001, versus 9N‐loaded saline‐treated controls. * = *P* < 0.05; *** = *P* < 0.001, versus baseline. The color of the symbol indicates the relevant treatment group.

For pharmacologic validation of the mechanical joint loading model, animals were loaded at 9N. Nonloaded saline‐treated animals showed no change in nociceptive thresholds over time, while, as in the experiments described above, the loaded saline‐treated group exhibited mechanical hypersensitivity and altered weight bearing from 2 weeks postloading (Figure 4). Both gabapentin and diclofenac relieved mechanical hypersensitivity (Figure 4A) and the altered weight bearing (Figure 4B) after 2 weeks of treatment, and gabapentin was more effective than diclofenac. After 2 weeks of gabapentin treatment, the mechanical threshold was increased (mean ± SEM 1.234 ± 0.11 gm) compared to baseline (0.148 ± 0.03 gm), indicating a significantly higher mechanical sensitivity than that observed in loaded controls (*P* < 0.001) (Figure 4A). In comparison, diclofenac increased baseline mechanical thresholds (0.083 ± 0.02 gm) after 2 weeks of treatment (0.472 ± 0.09 gm) when compared to loaded controls (*P* = 0.0057) (Figure 4A). Altered weight bearing was reversed after 2 weeks of treatment, and the percentage of weight borne on the ipsilateral paw increased with gabapentin (46.62 ± 3.0%) and diclofenac (47.23 ± 2.1%), compared to loaded saline‐treated controls (39.91 ± 2.1%) (Figure 4B).

**Figure 5:**
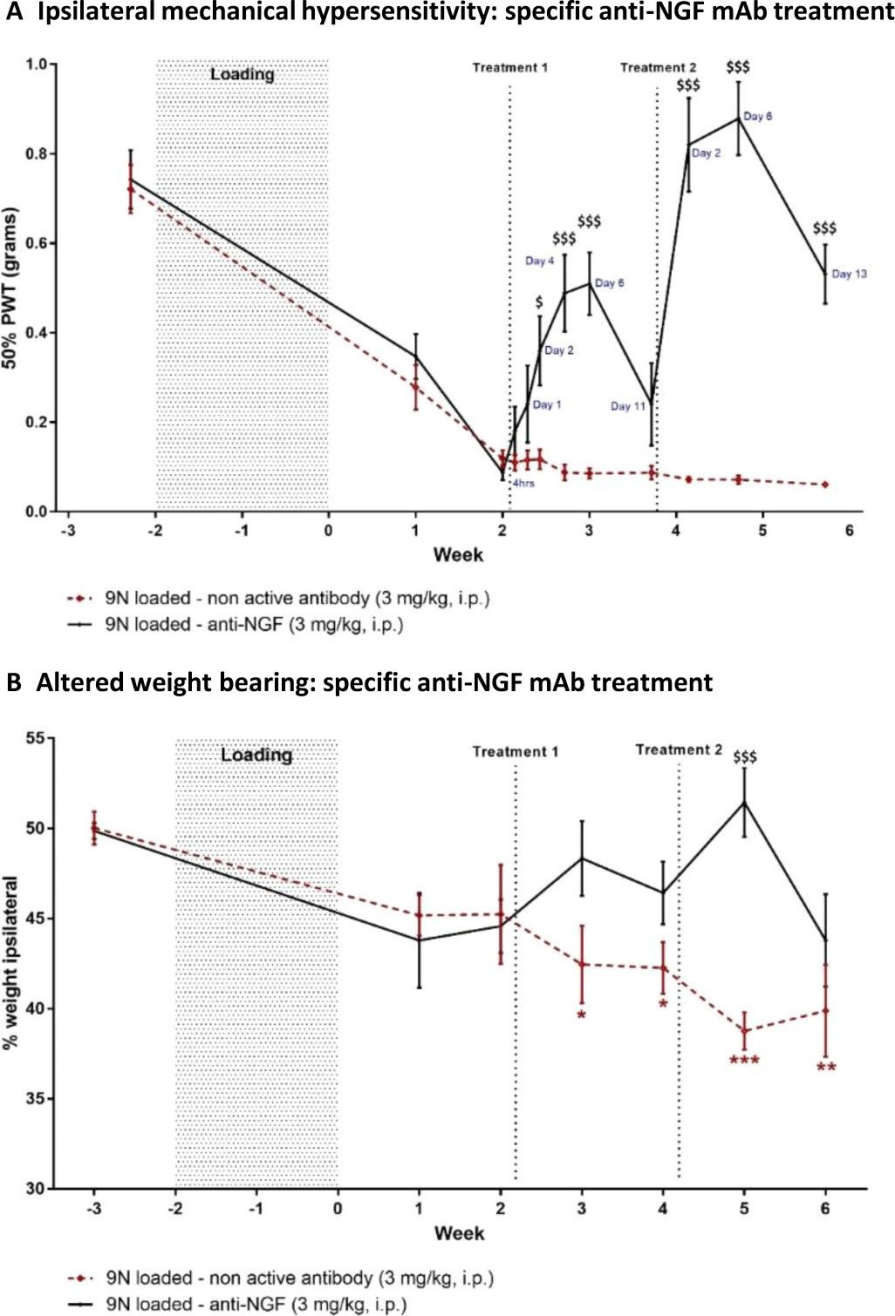
Effect of anti–nerve growth factor (anti‐NGF)monoclonal antibody (mAb) treatment on postloading mechanical hypersensitivity and altered weight bearing. Animals received anti‐NGF mAb treatment 2 and 4 weeks postloading. Mechanical hypersensitivity was monitored initially on a weekly basis and then on a more frequent basis after animals started receiving anti‐NGF (n = 8) or inactive antibody (n = 8) intraperitoneally (IP). **A**, 50% paw withdrawal threshold (PWT) was measured at the times (hours or days posttreatment) indicated. **B**, Weight bearing was monitored on a weekly basis and measured as the percentage of weight borne on the ipsilateral hind paw. Values are the mean ± SEM. $ = *P* < 0.05; $$$ = *P* < 0.001, versus controls. * = *P* < 0.05; ** = *P* < 0.01; *** = *P* < 0.001, versus baseline. Color figure can be viewed in the online issue, which is available at http://onlinelibrary.wiley.com/doi/10.1002/art.40835/abstract.

The first injection of anti‐NGF antibody effectively alleviated loading‐induced pain behaviors in treated animals (Figure 5), while the second injection showed a stronger and more prolonged analgesic effect. Two days after the first anti‐NGF mAb treatment, mechanical hypersensitivity was significantly alleviated in treated animals (0.360 ± 0.08 gm), compared to inactive antibody–treated animals (0.117 ± 0.02 gm) (*P* = 0.028). This effect lasted for 4 days, after which it dwindled. The second treatment with anti‐NGF mAb was also efficacious by 2 days postinjection, inducing a cumulative effect on mechanical sensitivity, which returned to and then exceeded baseline values (0.820 ± 0.10 gm), compared to controls (0.072 ± 0.01 gm) (*P* < 0.001). Efficacy of the second anti‐NGF mAb treatment lasted up to 13 days postinjection (Figure 5A). A similar pattern was observed for the effect of anti‐NGF mAb on weight bearing, as weight distribution was restored 1 week postinjection but efficacy was lost 2 weeks later (Figure 5B). A week after the second anti‐NGF mAb treatment, treated animals showed significantly improved weight bearing (51.44 ± 1.9%) compared to control animals (38.76 ± 1.0%) (*P* < 0.001) (Figure 5B).

**Figure 6:**
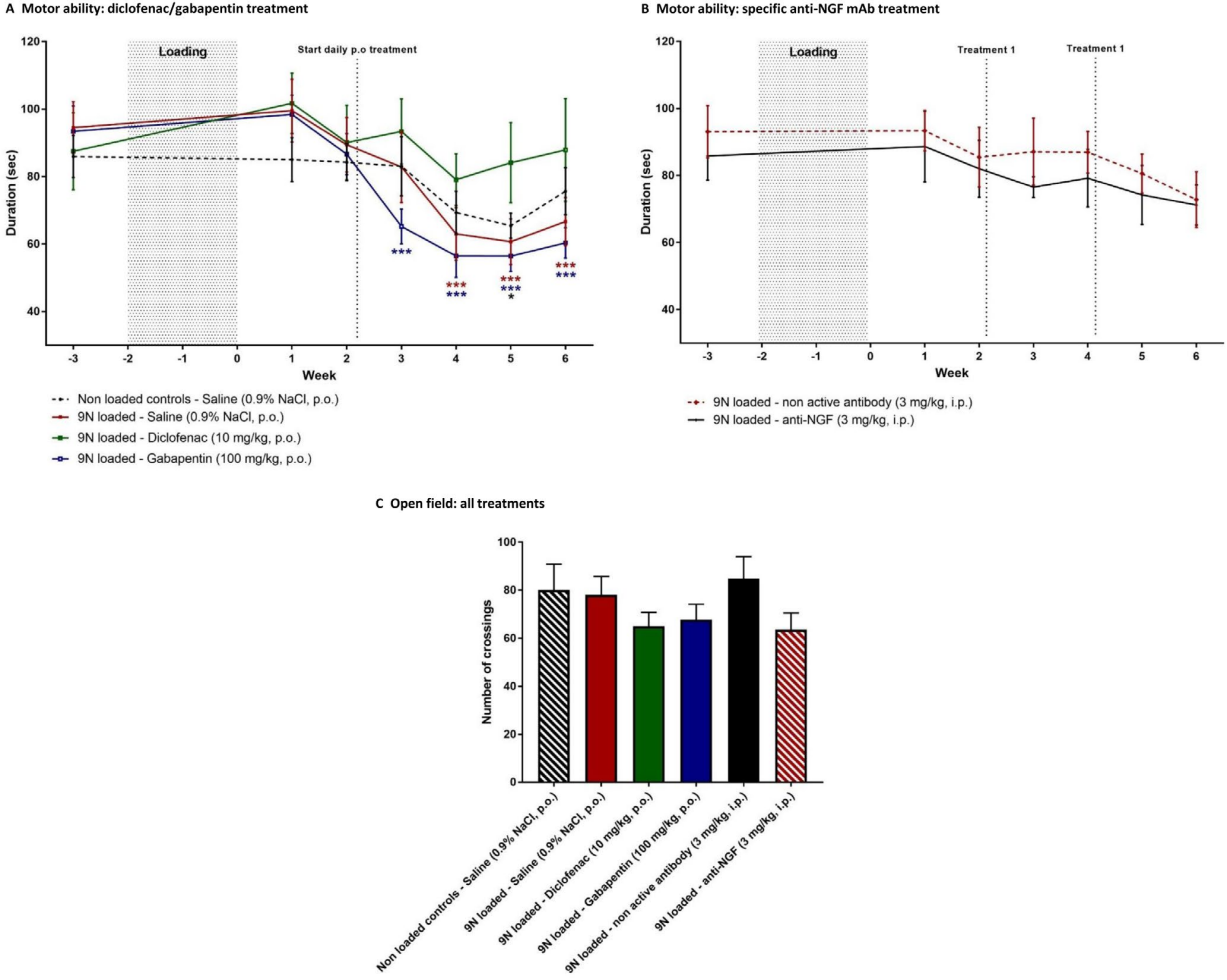
Effect of analgesic treatment on postloading motor ability and natural exploratory behavior. Motor activity was assessed as the duration mice were able to remain on the rotarod. **A**, Loaded controls (n = 8) and nonloaded controls (n = 8) received saline. Loaded treated animals received diclofenac (n = 6) or gabapentin (n = 8) orally (PO). **B**, Loaded animals received anti–nerve growth factor (anti‐NGF) (n = 8) or inactive antibody (n = 8) intraperitoneally (IP). * = *P* < 0.05; *** = *P* < 0.001, versus baseline. The color of the symbol indicates the relevant treatment group. **C**, Natural exploratory behavior, as assessed by allowing mice to cross in the open field for 5 minutes, was unaffected by treatment. Values are the mean ± SEM.

All treatment groups showed a similar decline in mobility, as measured by the rotarod, compared to both loaded control groups (saline‐treated and inactive antibody–treated mice) (Figures 6A and B), except for diclofenac‐treated animals, which did not show a decrease in time spent on the rotarod (Figure 6A). Furthermore, exploratory behavior was the same in all groups (Figure 6C), and none of the treatments influenced weight gain (data not shown).

## Discussion

In this study, we demonstrated that mechanical joint loading is an appropriate model to study mechanically induced OA pain. We characterized the symptomatic aspects of mechanically induced OA by measuring the development of nociceptive behavior in the presence of histopathologically evident OA. Furthermore, the first step was taken in validating the mechanical joint loading model by showing alleviation of nociceptive behavior when treated with different classes of analgesics.

Mechanical joint loading is known to induce alterations in articular cartilage 35, which, in cases of repetitive or excessive loading, can lead to OA 36. The mechanical joint loading model has been developed to explore the mechanisms responsible for mechanically induced OA 20. It mimics structural changes typically seen in human OA, such as spontaneously progressing articular cartilage lesions, subchondral bone changes, and osteophyte formation 20, 21. The noninvasive nature of this model has the added benefit of enabling examination of whole‐joint pathology in an intact knee. This avoids complications typical in surgical interventions, including postsurgery pain and infection risk, thus improving animal welfare and reducing variance in behavioral measurements.

In the first measurement of pain behaviors in this model, we found that mechanical joint loading at both 9N and 11N induced mechanical hypersensitivity, accompanied by altered weight bearing and reduced mobility, without affecting thermal sensitivity. The development of pain‐like behaviors was comparable at both loading intensity levels, with ipsilateral mechanical hypersensitivity and altered weight bearing developing 2 weeks postloading and contralateral mechanical hypersensitivity (as well as reduced mobility) developing 4–5 weeks postloading. This pain phenotype is similar to the pain observed in OA patients, which initially presents with hypersensitivity of the affected joint and pain during weight bearing. Frequency, duration, and severity of pain worsen as OA progresses, and peripheral as well as central neurologic mechanisms are recruited, which leads to centralized allodynia common in late‐stage OA 37. Consequently, the contralateral mechanical hypersensitivity observed after mechanical joint loading could be due to altered gait 21, which occurred when mice relieved ipsilateral hypersensitivity by compensating with their contralateral limb; alternatively, it could indicate a centralized hypersensitivity. No significant changes in behavioral measurements were observed in the first week after loading, which indicates that progressive mechano‐adaptive changes over time, rather than the initial insult of mechanical loading, were responsible for nociceptive behavior. Further studies showed that the initial cartilage lesions induced by mechanical joint loading at 9N worsened over time, suggesting the progressive nature of the development of nociceptive behavior (see Supplementary Data, http://onlinelibrary.wiley.com/doi/10.1002/art.40835/abstract). Taken together, these findings suggest that mechanical joint loading induces a nociceptive phenotype typical of progressive mechanically induced OA.

This nociceptive phenotype observed after mechanical joint loading is more comparable to surgical models of OA than to the MIA model. A stark increase in mechanical hypersensitivity and altered weight bearing immediately after injection, which can persist up to 28 days postinjection 8, 12, 38, is typically seen in the MIA model. In contrast, the mechanical joint loading model did not produce this immediate nociceptive response that is typical in the inflammatory form of OA. Furthermore, MIA injections in mice have not been shown to reduce motor ability 12, 38 or any contralateral nociceptive behavior. In the DMM model, mechanical hypersensitivity developed 2–4 weeks postsurgery and lasted up to 16 weeks 18, with altered weight bearing taking up to 12 weeks to develop 16 and no change in locomotion or thermal sensitivity 39. Although the onset of nociceptive behaviors appeared earlier in the mechanical joint loading model, the delay in behavioral responses seen in both mechanical joint loading and DMM models is common for a progressive form of OA. Additionally, DMM has been shown to induce contralateral nociceptive behaviors 39, comparable to those seen in the mechanical joint loading model, indicating compensatory behavior or central hypersensitization. In contrast, the mechanical joint loading model did not show any of the postsurgical pain or hypersensitivity in sham controls that is typical in surgical models 5, 17. Rather than relying on inflammatory damage of the joint as shown in the MIA model, both the mechanical joint loading and DMM models rely on a mechanical disruption and joint destabilization similar to that seen in human OA, in which excessive use or trauma leads to progressive joint damage.

A general drawback of this model is that there is no sham procedure that can control for or rule out off‐target damage induced by the loading procedure. The nonloaded controls do not get loaded but are subjected to the anesthesia procedure and, consequently, function as behavioral controls rather than controls for knee pathologies unrelated to mechanically induced OA. Mice loaded statically at 2N showed mild ipsilateral mechanical hypersensitivity that was neither consistent nor progressive (data not shown). Additionally, these mice exhibited mild ipsilateral lesions in the articular cartilage. This makes the 2N‐loaded mice inappropriate as controls for OA pain.

At 6 weeks postloading, both 9N‐ and 11N‐loaded mice exhibited lesions in the articular cartilage of ipsilateral and contralateral knees, confirming that mechanical joint loading induces an OA‐like histopathologic phenotype. Analysis of articular cartilage lesions at 1, 3, and 6 weeks postloading at 9N (see Supplementary Data, http://onlinelibrary.wiley.com/doi/10.1002/art.40835/abstract) showed that lesions in this study were comparable to those described by Poulet et al 20 at 3 weeks postloading in mice with the same loading regimen. Additionally, these results confirm the spontaneous exacerbation of lesions at 3 weeks postloading, compared to lesions seen directly after loading. The time frame in which these lesions progressed and worsened corresponds to the development of nociceptive behaviors in this model, suggesting that the progressive degradation of the knee induces this behavior. Furthermore, both 9N‐ and 11N‐loaded mice showed mild contralateral damage, which could explain the development of contralateral mechanical hypersensitivity seen in these animals.

Notably, 11N‐loaded mice had extensive ipsilateral damage, with lesions consistently reaching maximum scores, while 9N‐loaded mice showed milder OA histopathology without heightened nociceptive behavior. This implies that although cartilage damage is an important indicator of OA, it does not necessarily relate to the severity of pain. Pro‐osteogenic changes in the tibia, for which this model was originally developed, are typically seen only at loading magnitudes of 13N or higher 19. With loading regimens of 9N or 11N, no such osteogenic effects were observed (data not shown), indicating that bone remodeling of the tibia does not contribute to the mechanical joint loading–induced development of nociceptive behavior. Knee OA is a whole‐joint disease and, in patients with OA, moderate correlations between pain severity and tissue damage seen by magnetic resonance imaging or radiography have been shown for a variety of knee tissues, including joint space narrowing 40, subchondral bone changes, synovitis, and meniscal tears 41. Additional experiments will be needed to study the effect of mechanical joint loading on other joint tissues and to identify their role in the development of nociceptive behavior. The lack of difference in pain profile seen between the 9N‐ and 11N‐loaded mice could reflect the modest sensitivity of pain parameters used, all of which are measurements for referred pain. However, findings from our study clearly show that 11N‐loaded mice develop the maximum possible knee damage, thus reaching a ceiling effect in both OA severity score and pain phenotype. The severe knee pathology seen in these mice could indicate that mechanical joint loading at 11N induces damage that is not restricted to the cartilage but also affects other joint tissues. Combined with the observation that the 9N‐loaded mice develop a milder form of OA but still show a robust pain phenotype, it was concluded that a 9N loading regimen was more appropriate for follow‐up pharmacology studies.

Diclofenac, gabapentin, and anti‐NGF mAb, analgesics used to treat OA pain in patients, were effective in alleviating mechanical joint loading–induced nociceptive behavior. Additionally, these treatments had no effect on the exploratory behavior or weight of the mice, demonstrating that animal welfare was not compromised. We also showed that none of these treatments compromised mobility, suggesting that the restoration of behavioral responses to baseline values was due to their analgesic effects rather than possible sedative side effects or motor impairment.

In the first 2 weeks of treatment, gabapentin was more effective in alleviating mechanical hypersensitivity and restoring weight bearing than diclofenac. This is particularly striking considering that diclofenac, which is typically effective in treating inflammatory pain, is the first‐line treatment for OA, while gabapentin is more commonly used to treat neuropathic pain. Despite the preferential effectiveness against neuropathic pain, gabapentin has been shown to be effective in treating nociception in both MIA models 42 and surgical models 43, 44 of OA. The demonstrated efficacy of gabapentin in several OA pain models suggests that OA pain could be, in part, neuropathic. In fact, in the PMM model of OA, diclofenac was effective only in treating nociception in the initial inflammatory phase but not at a later stage, whereas gabapentin alleviated the mechanical hypersensitivity seen in the chronic phase of OA‐induced nociception 5. Taken together, these findings suggest that although inflammation and the resulting pain likely play a role in OA pathology, OA is a complex pain syndrome with a significant neuropathic component.

Anti‐NGF antibody treatment resulted in a prolonged and significant reduction in nociceptive hypersensitivity, with repeated treatment increasing the magnitude and duration of its effectiveness. There is a lot of evidence supporting the role of NGF in OA pain 45. Chondrocytes produce NGF in response to degeneration, NGF levels are elevated in the synovial fluid of patients with OA, and in clinical trials anti‐NGF mAb treatment has provided significant pain relief in OA patients 46. Furthermore, in the MIA and medial meniscal transection murine models of OA, intraarticular injections of NGF increased nociceptive behavioral responses in both experimental and healthy control animals, suggesting that NGF plays a role in the severity of OA pain 47. The prolonged effectiveness of anti‐NGF mAb treatment in the mechanical joint loading model is similar to that in the MIA model, in which anti‐NGF effectively restored altered gait for up to 35 days posttreatment 30, 48.

Historically, several murine models have been useful in elucidating mechanisms of the pathogenesis of OA. The ease of genetic modification, relatively low costs, and reduced time needed for disease progression mean that mice are widely used in both OA and pain research. Here, we present an alternative model that closely mimics an OA pain phenotype that is typical in mechanically induced OA. Our findings show that the noninvasive mechanical joint loading model induces OA lesions and generates a reproducible pain phenotype that can be reversed using known analgesics for OA pain, thus suggesting its utility as an alternative model to study OA pain.

## Author Contributions

All authors were involved in drafting the article or revising it critically for important intellectual content, and all authors approved the final version to be published. Dr. ter Heegde had full access to all of the data in the study and takes responsibility for the integrity of the data and the accuracy of the data analysis.

### Study conception and design

Ter Heegde, Luiz, Chessell, Welsh, Wood, Chenu.

### Acquisition of data

Ter Heegde, Luiz, Santana‐Varela.

### Analysis and interpretation of data

Ter Heegde, Luiz, Wood, Chenu.

## Additional Disclosures

Authors Chessell and Welsh are employees of AstraZeneca.
