# Supplementary material for: Noninvasive Mechanical Joint Loading as an Alternative Model for Osteoarthritic Pain
Source: Arthritis Rheumatol. 2019 May 17;71(7):1078–88. doi: 10.1002/art.40835 (PMC6618037; doi:10.1002/art.40835)
Supplement: Supplementary file 1 [file ART-71-1078-s001.docx]

## Supplementary methods: measurement of pain-associated behaviours

Animals were trained and allowed to acclimatize to the behavioural testing set-up prior to the start of the experiment. Behavioural experiments were done in a separate room to ensure a quite environment during testing. For each time point, non-thermal behavioural testing was done on the same day with mechanical hyperalgesia being tested first, followed by weight bearing and lastly by motor ability testing. Between each behavioural assay, mice were allowed a minimum of an hour rest. Thermal assays were done on separate days. Researchers performing behavioural testing were blinded to the condition of the mice but not to the week of the experiment. For each behavioural assay the same researcher followed the animals for the duration of the experiment. Researchers conducting both the MJL to induce OA and the following behavioural testing were female.

#### Mechanical hyperalgesia

Mechanical hyperalgesia was assessed by measuring touch perception thresholds with nylon von Frey microfilaments (Bioseb). The up-down method was used for obtaining 50% paw withdrawal threshold as described previously [1, 2]. Briefly; the 0.4g filament was always used to start the sequence, if a positive response was observed, a filament of a lesser weight would be used whilst if no response was observed then a filament of a higher weight would be employed. This was repeated until 6 responses had been noted from which 50% paw withdrawal threshold could be calculated. Mechanical sensitivity thresholds were measured in both hind paws, first in ipsilateral paws then contralateral after a 30-minute rest.

#### Weight bearing asymmetry

Static weight bearing was used to assess the amount of weight borne on the ipsilateral leg [3]. Mice were positioned on an incapacitance meter (Linton Incapacitance Tester) consisting of two scales with each hind limb placed on one of the scales and front limbs resting on the holding chamber. Once mice were still and resting, weight placed on each of the scales was measured for 5 seconds. Measurements were taken in triplicate allowing for the mice to readjust between measurements. Weight-bearing ratio, weight placed on affected limb divided by the total weight placed on both hind limbs, was calculated for each trial and then averaged per time point.

#### Motor ability

Motor ability was measured using the rotarod test (IITC, mouse-adapted apparatus) over a period of 5 minutes [4]. Prior to baseline testing, mice were trained on the rotarod for three consecutive days. Initial starting ramp was set at 4rpm which was increased steadily up to 40rpm at 3 minutes and remained at 40rpm for the last 2 minutes. Duration that mice were able to stay on the rotarod was measured. Measurements were taken in triplicate and averaged for each time point.

#### Exploratory behaviour

Open field test was used to observe the spontaneous exploration motor activity of mice [5]. Each mouse was placed in one of the four corner squares facing the wall and allowed to freely explore the environment for 5 min. The total number of times of crossing in the open field was recorded as a measure of spontaneous pain [6].

#### Thermal hypersensitivity

Nociceptive heat threshold was determined by measuring hind paw withdrawal latency using the Hargreaves apparatus (IITC) at a ramp of 1.5⁰C per second with a 30 second cut-off [7] as well as the latency for nociceptive behaviour on a hotplate (Ugo Basile) at 50⁰C and 55⁰C with a cut-off at 60 seconds [8]. Cold thermal hypersensitivity was measured using cold plantar assay (dry ice model according to [9]) and by measuring the latency for nociceptive behaviour on a cold plate (Ugo Basile) at 0⁰C with a cut-off at 60 seconds [8].

## References

1. Chaplan, S.R., et al., *Quantitative assessment of tactile allodynia in the rat paw.* J Neurosci Methods, 1994. **53**(1): p. 55-63.

2. Bonin, R.P., C. Bories, and Y. De Koninck, *A simplified up-down method (SUDO) for measuring mechanical nociception in rodents using von Frey filaments.* Mol Pain, 2014. **10**: p. 26.

3. Malfait, A.M., C.B. Little, and J.J. McDougall, *A commentary on modelling osteoarthritis pain in small animals.* Osteoarthritis Cartilage, 2013. **21**(9): p. 1316-26.

4. Jones, B.J. and D.J. Roberts, *The quantiative measurement of motor inco-ordination in naive mice using an acelerating rotarod.* J Pharm Pharmacol, 1968. **20**(4): p. 302-4.

5. Hall, C. and E.L. Ballachey, *A study of the rat’s behaviour in a field: a contribution to method in comparative psychology.* University of California Publications in Psychology, 1932. **6**(1-12).

6. Tappe-Theodor, A. and R. Kuner, *Studying ongoing and spontaneous pain in rodents--challenges and opportunities.* Eur J Neurosci, 2014. **39**(11): p. 1881-90.

7. Hargreaves, K., et al., *A new and sensitive method for measuring thermal nociception in cutaneous hyperalgesia.* Pain, 1988. **32**(1): p. 77-88.

8. Eddy, N.B. and D. Lembach, *Synthesis, Anti-Inflammatory and Analgesic Activity of Pyrido [2, 1-b] Quinazoline Derivatives.* J Pharma Exp, 1953(953): p. 703-385.

9. Brenner, D.S., J.P. Golden, and R.W.t. Gereau, *A novel behavioral assay for measuring cold sensation in mice.* PLoS One, 2012. **7**(6): p. e39765.
