# Supplementary material for: Noninvasive Mechanical Joint Loading as an Alternative Model for Osteoarthritic Pain
Source: Arthritis Rheumatol. 2019 May 17;71(7):1078–88. doi: 10.1002/art.40835 (PMC6618037; doi:10.1002/art.40835)
Supplement: Supplementary file 2 [file ART-71-1078-s002.docx]

## Supplementary data: progression of histological changes after MJL at 9N

Additional experiments were conducted to track knee pathology following MJL at 9N to verify that the articular cartilage lesions induced by the initial insult of the MJL would worsen progressively over time.

Briefly, naïve male 12-week-old C57/BL6 mice were loaded at 9N for two weeks to induce OA after which they were sacrificed at one, three or six weeks post loading. Non-loaded, anaesthetised age and cage matched, controls were sacrificed at the same time points. Both hindlimbs were collected immediately after sacrifice, processed for paraffin embedding, and sectioned and stained for OA grading. A quarter of the sections were used for the OA grading while remaining slides were subjected to standardized haematoxylin and eosin staining in order to visualize synovial thickening.

Results showed that, following MJL at 9N, the severity of articular cartilage damage increases over time matching the progressive nature in the development of nociceptive behaviour (Figure 1). Furthermore, although there is evidence of a thickening of the synovial lining at week one post-loading this is no longer evident at three and six weeks post-loading (Figure 2). This could suggest that these initial joint adaptions are not directly responsible for the pain phenotype as mice loaded at 9N do not show significant behavioural changes indicative of pain in the first week post-loading. It should be noted, however, that articular cartilage damage is only one measurement of OA and that other tissues of the joint contribute to the knee pathology seen in OA. Although the progression of cartilage damage matches that of the pain phenotype, further experiments are needed to study the role of other joint tissues in the development of both the knee pathology and nociceptive behaviour.

| 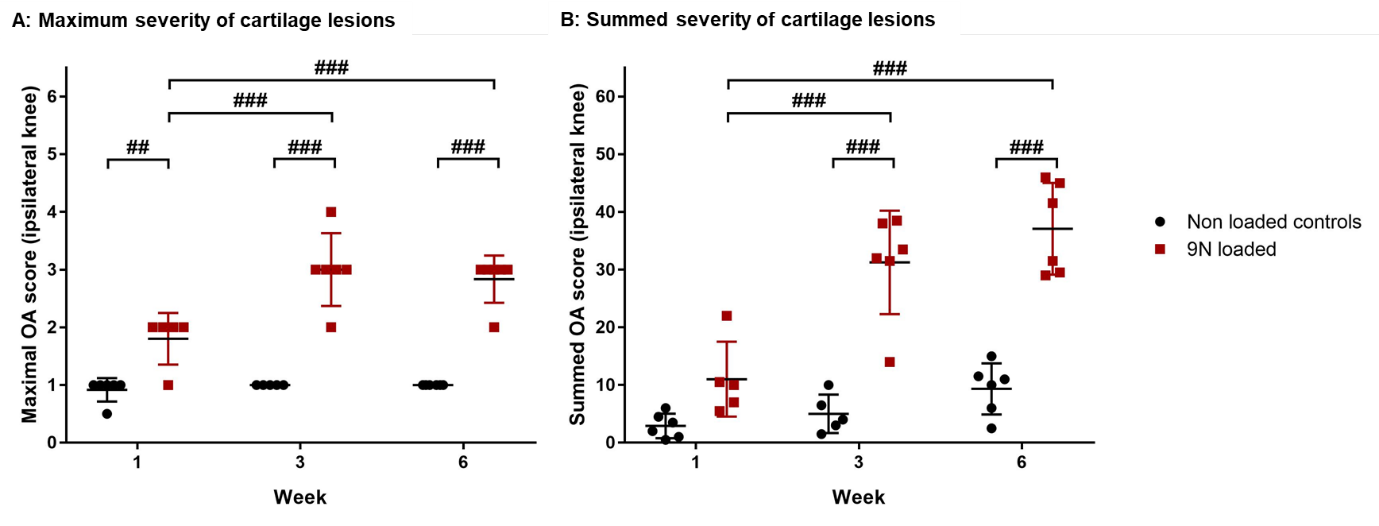  Figure 1 Severity of OA lesions at one, three and six weeks after MJL at 9N.  Ipsilateral knees of non-loaded and 9N-loaded mice were collected post mortem at 1,3 and 6 weeks post-loading and OA severity was scored (scoring system from 0-6, OA severity is classified as either low (grade 0-2), mild (grade 3-4) or severe (grade 5-6)). Maximum OA scores (**A**) and summed OA scores (**B**) are given for non-loaded (black circles, *n* = 6) and 9N-loaded mice (red squares, *n* = 6). Significant differences in the severity of OA lesions indicated with a ## (*p* < 0.01) or ### (*p* < 0.001). Values given as mean ± SEM. |
| --- |

| 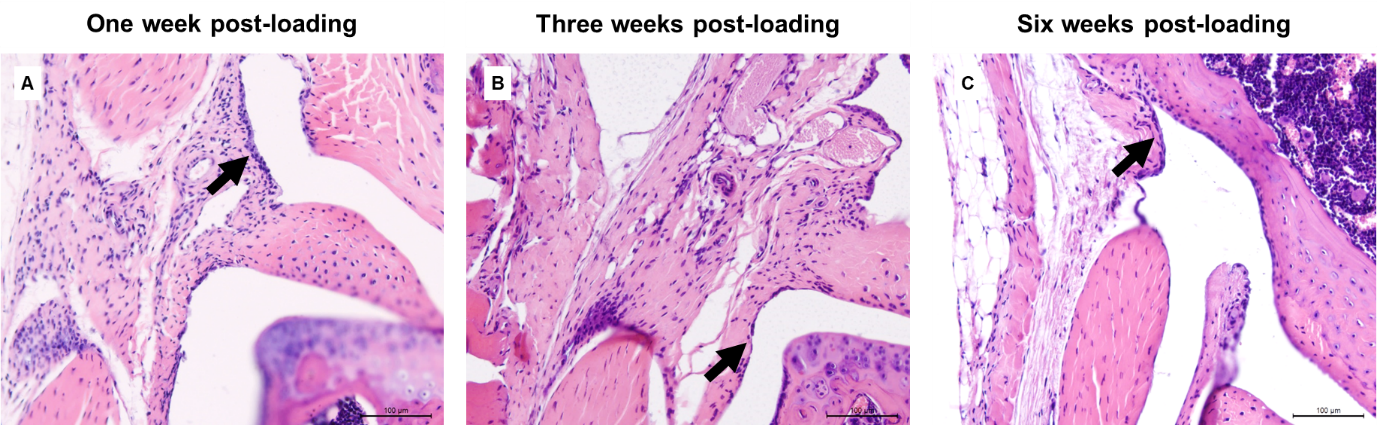  Figure 2 Decrease in thickening of synovial lining at one, three and six weeks after MJL at 9N  Coronal knee section of 9N-loaded mice, sacrificed at one (**A**), three (**B**) and six (**C**) weeks post-loading, were stained with haematoxylin and eosin to visualize the thickness of synovial lining as the MJL-induced OA progresses. Arrows indicate the synovial lining used to judge thickness. Images are shown at 20x magnification. |
| --- |
